# Uses of Molecular Docking Simulations in Elucidating Synergistic, Additive, and/or Multi-Target (SAM) Effects of Herbal Medicines

**DOI:** 10.3390/molecules29225406

**Published:** 2024-11-16

**Authors:** Sean P. Rigby

**Affiliations:** Department of Chemical and Environmental Engineering, Faculty of Engineering, University of Nottingham, University Park, Nottingham NG7 2RD, UK; sean.rigby@nottingham.ac.uk

**Keywords:** molecular docking, systems biology, herb, network

## Abstract

The philosophy of herbal medicines is that they contain multiple active components that target many aspects of a given disease. This is in line with the recent multiple-target strategy adopted due to shortcomings with the previous “magic bullet”, single-target strategy. The complexity of biological systems means it is often difficult to elucidate the mechanisms of synergistic, additive, and/or multi-target (SAM) effects. However, the use of methodologies employing molecular docking offers some insight into these. The aim of this work was to review the uses of molecular-docking simulations in the detection and/or elucidation of SAM effects with herbal medicines. Molecular docking has revealed the potential for SAM effects with many different, individual herbal medicines. Docking can also improve the fundamental understanding of SAM effects as part of systems biology approaches, such as providing quantitative weightings for the connections within static networks or supplying estimates of kinetic parameters for dynamic metabolic networks. Molecular docking can also be combined with pharmacophore modeling in a hybrid method that greatly improves the efficiency of screening. Overall, molecular docking has been shown to be a highly useful tool to provide evidence for the efficacy of herbal medicines, previously only supported by traditional usage.

## 1. Introduction

### 1.1. Synergistic, Additive, and/or Multi-Target (SAM) Effects

The overall aim of drug discovery is to obtain highly effective and safe drugs, with a low level of undesirable or toxic side effects [1]. The introduction, and more widespread use, of the target-based strategy for drug discovery seems to have coincided with a period of steady decline in the productivity of new drugs [2]. The target-based strategy usually involved identifying, based upon genetic analysis or biological observations, that a single gene, gene product, or molecular mechanism (e.g., a particular enzyme) was the underlying basis of a disease state and then targeting it specifically. This approach did have some early notable successes. However, many disease states are generally much more complicated than this and involve multiple processes occurring at one, or more, of the gene, cell, organ, and organism levels. This is because evolution has generated deeply integrated biological systems with many feedback interactions between levels [3]. Supposed single-target drugs may also not have just the anticipated effect due to interactions between various pathways in the disease network and unexpected promiscuous binding to many targets [4,5]. Large-scale gene knock-out experiments in model organisms have shown that, due to the evolved redundancy in biological networks making them resistant to even strong perturbation or interruption at single points, interventions may actually be required at multiple sites to achieve the desired overall effect [3,6,7]. This is because, as will be seen below, there are often compensatory signaling (or other) routes that bypass the inhibition of single-target proteins [8]. These considerations help to explain the recent failures of the single-target-based strategy and highlight the need for alternative strategies to find effective drug-based therapies.

One such alternative strategy is the “multi-target” therapeutic concept whereby multiple sites within a biological system are targeted together [1]. Further, while unintended interactions with other drugs was typically seen as detrimental to the single “magic bullet” compound approach of the single-target strategy, the multi-target approach can employ a mixture of various different compounds whose effects are intended to interact.

The key characteristic of herbal medicines is that they contain an, often, complex mixture of active compounds. The multi-target approach is, thence, consistent with the underlying philosophy of herbal medicines because the efficacy of herbal medicines is thought to be due to the action of many components with a weak to medium biological activity and with that activity toward a range of targets [9]. Obviously, there is still a need to check for side effects.

The various, underlying mechanisms of interaction between components of the mixture may involve a variety of processes. For example, one compound can be protected from degradation by enzymes through the presence of another substance inhibitory to the same enzymes [1]. Alternatively, one substance can modify the transport of another substance across a key barrier, such as various cell membranes. In addition, one compound may act upon a signaling system within the host’s cells that results in the changed efficacy of another compound compared to the latter alone [1]. The necessity for the various interactions of many compounds within a mixture, making up a herbal medicine, to achieve an overall therapeutic effect may explain why the conventional single-target strategies that fractionate and individually test the various components of a herbal mixture often fail to find any significant evidence of the therapeutic effect claimed on the basis of the traditional use of the herb [10].

The overall observational consequences of drug interactions are classified using a number of terms, including potentiating, additive, synergistic, and antagonistic effects [1]. Efferth and Koch [1] defined these observed effects as follows: “Positive interactions that enhance the potency of a bioactive compound by an inactive adjuvant substance” are called “potentiation”. If the “combined outcome [of two or more drugs] is equal to [just] the sum of the effects of the individual components”, then this is denoted as an “additive” effect. However, when such a combination of bioactive components results in “an effect that is greater than the sum of [that for] the different substances”, this is known as a “synergistic” effect. In addition, Spinella [11] proposed that the interactions of a synergistic type can be further sub-divided into two sub-types depending on the nature of the interaction, namely, pharmacodynamic or pharmacokinetic. For example, pharmacodynamic synergy results from combinations of allosteric modifiers at the gamma-aminobutyric acid A (GABA) receptor, while pharmacokinetic synergy results from interactions during the processes of drug absorption, distribution, biotransformation, or elimination [11]. Conversely, where the effect of the combination of interactions is less than the additive effect of the individual components, this is called “antagonism”. This work will concentrate on synergistic and additive interactions involving multi[ple] targets, according to the definitions above, henceforth referred to as SAM effects. However, it is noted that some differences in definitions for the above terms do exist in the literature [1]. In addition, negative interactions called “interferences” can also be of a pharmacokinetic type if they reduce the stability or bioavailability, or increase the metabolism, of the bioactive compound [6].

**Figure 1:**
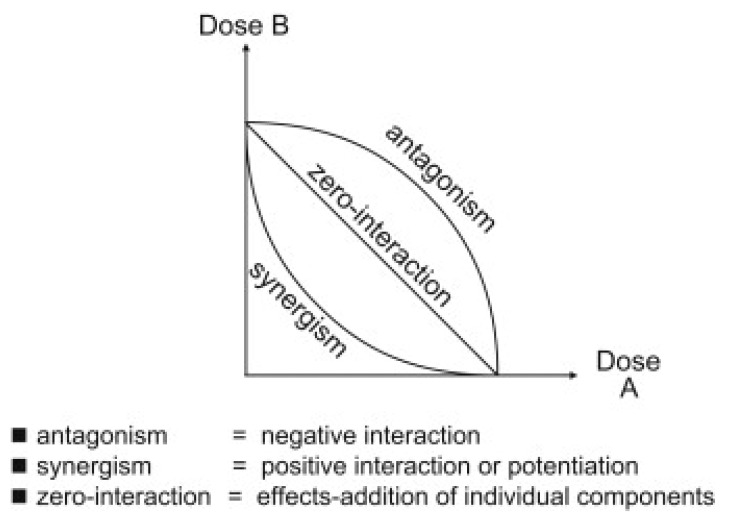
Isoboles for zero interaction (additive), synergism, and antagonism. Reprinted with permission from Ref. [14]. Elsevier, 2009.

A common method to highlight drug interactions experimentally is to construct an isobologram [12], as seen in Figure 1. This involves plotting, as *x* and *y* co-ordinates, the potential combinations of the individual doses of two drugs that result in a desired overall effect level. If the interaction between the two drugs is purely additive, then joining up the aforementioned points will result in a contour (the “isobole”) that is a straight line; but, if the interaction is synergistic, the contour will have a concave form (i.e., lie below the straight line on the plot joining the two points on the axes corresponding to the respective doses of just one of those drugs that achieves the desired effect). Conversely, if the interaction is antagonistic, the contour will have a convex form. However, this method, and similar ones, that only consider “two-dimensional” interactions cannot be used to study complex mixtures with multiple components. An alternative experimental approach that can cope with multi-component mixtures involves conducting biological assays or clinical studies that compare the outcomes from administering the same dose of an isolated, individual component on its own, versus the mixture, from which it comes, as a whole [12]. If the response is different in the two cases, then a SAM effect may be occurring. However, a suitable assay may not be available, and such experimental studies may be too expensive. Further, this type of study tends not to reveal the mechanistic reason for any effect. In contrast, in silico methods, such as molecular docking [13], can be much cheaper, more readily available, and also, potentially, reveal more concerning the mechanism of a SAM effect. Hence, this work will consider molecular-docking approaches to finding and studying SAM effects.

### 1.2. Systems Biology Approach

The increasing consciousness of the problems with the single-target strategy has led to the development of the so-called “systems biology” approach [15,16]. The aim of systems biology is “to understand physiology and disease from the level of molecular metabolic pathways, regulatory networks, cells, tissues, organs and ultimately the whole organism” [17,18]. Methodologies that consider systems biology approaches include networks (the modeling of flows and pathways within cellular networks using graph-like mathematical structures), systems modeling (modeling whole organ and tissue systems), cell modeling (the mathematical and computational simulation of whole cells), and target prioritization and drug development (applying the network, cell, and system models to target search and selection to assist the drug discovery process) [19]. These methodologies promise ways to make sense of, and discern useful structure in, the enormous, and thus otherwise overwhelming, genomic, proteomic, and dynamic datasets being generated via high-throughput techniques [18,19]. In particular, “omics” can be used to “ask what genes, proteins or phosphorylation states of proteins are expressed or upregulating”. Systems biology approaches can be classified into either “top-down” or “bottom-up”-type methods. Top-down methods, such as statistical analyses and static networks, are frequently applied to interpreting “omics” data in order to determine the underlying organization of a system or to mine information specific to a particular biological process. Alternatively, bottom-up methods, such as metabolic networks, model the continuous dynamic aspects of biological systems. These latter models thus require sufficient mechanistic knowledge and quantitative kinetic parameters not needed for the top-down models. Hence, the number of components that can be modeled with the bottom-up models is generally less than is possible with the top-down methods. As will be seen below, molecular docking can aid with constructing both types of methods.

Issues have arisen with developing systems biology approaches. It remains infeasible to produce exhaustive models fully integrating the molecular, cellular, organ, and organism levels due to limitations on current computing power [17]. Further, even if computing power was up to the job, the requisite level of information on the whole structure of the systems often does not exist yet. However, one practical way to still make progress in the face of these limitations is to adopt a modular approach and assume that the whole system can eventually be built up of constituent modules. Below, it will be seen what can be achieved using the de facto construction of a module for the arachidonic acid (AA) metabolic network in different types of cells. It will also be seen how molecular-docking simulations can supply the necessary model parameters that are not easily available via experimental measurements.

### 1.3. Aims and Objectives

The main aim of this work is to review the uses of molecular-docking simulations in the detection and/or elucidation of synergistic, additive, and/or multi-target (SAM) effects in herbal medicines. It will first describe the basic principles of molecular docking, and then it will go on to describe how molecular docking is incorporated into a common approach for screening for activity of multiple components of molecular mixtures from herbal medicines and which can detect SAM effects. It will then present a survey of examples from the literature where this approach has been applied to a range of disease states. This work will then discuss how molecular docking can be combined in more complex methodologies involving various types of network-based approaches from systems biology, and also be combined with pharmacophore methods, which can greatly help to understand the mechanism of SAM effects.

## 2. Molecular Docking

### 2.1. Basic Principles of the Docking Approach

Protein–ligand docking is now a widely used tool for drug discovery and is already described in great detail elsewhere [20,21], so only key relevant points will be summarized here. The “central dogma” of the docking approach “is that compounds that dock correctly into the receptor are more likely to display biological activity than those that do not dock” [20]. The application of docking requires the availability of sufficiently realistic representations of the relevant chemical entities that can be understood by a computer program. If a target structure and binding site are known, docking can be used to establish where, and how, exactly a ligand will bind and predict the strength of the interaction. Docking software generally utilizes protein structures, previously obtained via X-ray crystallography, downloaded from a database, such as the Protein Database (PDB). If the binding site of a protein is not known, it can be predicted based on the primary structure using homologous models of a known structure [13]. The database protein structure is then often cleaned (of other molecules included with the recorded structure). The docking algorithm itself typically involves the generation of an ensemble of 3D conformers of a complex starting from the known structures of its free components. The ways this is performed are reviewed extensively elsewhere [20,21] and so will not be covered in detail here. For protein–ligand docking, this involves searching through different conformations and orientations (known as the “pose”) of the ligand within the target protein and measuring the binding affinity corresponding to each alternative. The set of various poses to attempt is, itself, generated by an optimizer algorithm, which, ideally, should sample the complex search space made up of the degrees of freedom of the protein–ligand complex sufficiently exhaustively to include the true binding mode [20]. These degrees of freedom can be just the six degrees of translational and rotational freedom if the ligand and target are both treated as rigid bodies, or many more if either the ligand, or both ligand and target, are also allowed to be flexible. The greater the degrees of freedom, the greater the complexity of the search space and, then, the greater the demands on computational power. Machine learning methods, such as genetic algorithms, are often used to improve accuracy and to speed up the optimization process [22]. In order to identify the “true” pose, the various candidates must be evaluated and ranked by means of a scoring function of some sort that can distinguish even similar poses sufficiently to find the “true” binding mode. Scoring functions come in three major types, namely, force field based, empirical, and knowledge based [23], and must be fast in implementation to allow the rapid screening of many conformers [20]. Force-field-based scoring functions calculate interaction energies, incorporating terms such as van der Waals forces and electrostatic interactions. However, knowledge-based scoring functions use statistical potentials derived from contact frequencies. Empirical scoring functions estimate binding free energies by using several different terms, frequently using linear regression methods. However, the “best” combination of the search algorithm and scoring function, to identify the true pose, is often very system (ligand and target) specific. The choice of the detail of the physical descriptions and internal parameters for the algorithms can also affect the accuracy of the final “best” pose found. These are reviewed extensively elsewhere [20,21] and so will not be detailed here. More recently, Artificial Neural Network (ANN) techniques have also been used to automate and optimize the docking process, reducing the time and resources required for molecular docking [23]. Some docking methodologies use multiple algorithms in parallel to try to get a consensus, but this adds to computational time. Typically, the pose with the highest binding affinity is the one in which free energy is the most negative. However, there is a key difference, in the importance of accuracy and precision necessary, between the use of docking in single-target strategies and searching for herbal medicines with SAM effects. This is because, as has been mentioned above and will be discussed further below, herbal medicines are often composed of mixtures of both strong and weak ligands, or even just mixtures of compounds with only middling binding affinity. Hence, a high accuracy of binding affinity estimation is not so vital to distinguish definitively the paramount best hit(s).

### 2.2. A Common Screening Approach Involving Docking

A common basic approach has frequently been adopted in the literature for testing for, and/or understanding the mechanism of, synergistic and/or multi-target effects for herbal medicines. A particular herb, or herbs, is selected based upon traditional use, or previous suggestive findings in the scientific literature, for a certain disease state. The potential active molecules in the herb(s) are identified either from existing databases (e.g., those listed in [24,25]) or by chemical means (e.g., LC–MS analysis of the extract of the herb [26]). The set of targets for addressing the chosen disease state is identified from existing databases or via a literature search and the structure of the relevant binding site obtained from a database (e.g., PDC) or otherwise constructed, such as by homology modeling [26]. The set of test molecules may be pre-screened for “drug-like” character via the application of Lipinski’s rule of five. However, recent work suggests that good candidate molecules may be discarded based upon this rule [27,28]. Indeed, plants have provided successful oral drugs that violate the rule of five [29]. These molecules tend to be of high complexity, rich in stereogenic centers, and relatively lacking in nitrogen compared to synthetic drugs. These molecules may have been optimized by evolution to take advantage of active transport, while the rule of five only applies to compounds absorbed by passive mechanisms [29]. Furthermore, machine learning can also be used to determine pharmacokinetic profiles of molecules and extend the range of molecules accepted beyond those that meet the rule of five [30].

Each set of test molecules is then docked with each target to obtain the docking score. In order to get a consensus, to avoid false positives, often two or more docking tools are used in parallel, e.g., [31]. The synergistic and/or multi-target potential of a given herb is then assessed based upon the scores. The molecular-docking simulation is also often followed up with molecular dynamics simulations to validate the docking score. In the simplest version possible for this type of study, one molecule can be docked with one molecular target, such as in the study of jensenone from eucalyptus essential oil as a potential inhibitor of the main viral proteinase of COVID-19 [32]. However, this study does not consider additive or synergistic effects between herbal constituents. Since the basic principles as described above are often adopted in many studies, the results of these have been summarized in Table 1. Details of how these studies were selected are included in the Appendix A. However, the nature and depth of the reporting of the findings of molecular-docking studies varies, especially where large numbers of molecule-target pairs are involved. The study is classed as demonstrating an additive or synergy effect if such is explicitly claimed or was apparent from the published findings. Many docking studies now augment the findings with systems biology tools such as some form of network, and the use of such a tool is also recorded in Table 1. Some studies involving combined docking and network approaches will be discussed in more detail below.

Table 1 shows that molecular-docking studies have revealed that a variety of herbal medicines, when applied to a range of disease states, contain several active components. In some cases, multiple molecules from the mixture from one herb have affinity for the same target, and, in other cases, several different molecules have affinity for a range of targets related to a particular disease state. SAM effects have been detected in most cases. It has also been seen that there are a few studies where molecular docking has been combined with systems biology approaches, particularly static networks, as will be discussed in more detail below.

The basic molecular-docking study described above is often just a component of a much broader study that also involves experimental work. The docking can be used to suggest potential trial systems to validate via experiment, or the molecular docking can be used to elucidate the mechanism of a SAM effect already discovered via experiments.

It has been pointed out that virtual screening using simpler molecular-docking approaches has some issues [7,57]. Some of the issues highlighted in previous studies include limitations in the protein structures available in the PDB, a high false positive rate, difficulties in considering target flexibility, the inaccuracy of scoring functions for estimating target–ligand binding free energy, and the limitations in inferring the wider physiological impact(s) of a particular ligand–target binding.

Since the docking method requires the target protein structure, an alternative approach is needed when this is not available. For example, structure–activity analysis may be used for the prediction of biological activity and other properties of organic compounds based on their structural formulas [58].

Further, while virtual screening alone using molecular docking may have a low hit rate of only ~ 30% for initial hits, Wang et al. [59] have suggested that molecular-docking simulations can be used as a preliminary screen to determine candidate herbs to submit for more effective screening with experimental affinity mass spectrometry. The TCM database was used to identify 2920 compounds with known anti-tumor activity. This set of test compounds was screened using multiple docking software types to identify hits for the GTP-binding pocket involved in the GTPase activity of the Ras protein since this protein is an intracellular guanine nucleotide-binding protein that regulates cell proliferation, survival, differentiation and apoptosis. Analysis of the docking scores for the compounds showed that most of the high-scoring compounds came from 11 particular herbs, and scaffold cluster analysis showed that most of the high-docking-score compounds were isoamylene containing flavonoids and 20(s)-protopanoxadio saponins. Affinity MS screening was then used to verify that the related crude mixture of compounds derived from each herb had the expected affinity for the target protein. Ultimately, the affinity MS testing showed that, of 18 hits unique to the virtual screening, 11 of them could not be verified using the affinity MS technique and, thus, were likely to be false positives. However, the key structural aspects of the hit compounds identified in the virtual screening were confirmed by the affinity MS experiments.

### 2.3. The Reverse-Docking Approach

The so-called “forward docking” approach would be to screen many potential drug (ligand) compounds against each single target, whereas, the so-called “reverse docking” approach is to screen multiple potential targets against each ligand molecule [57], otherwise the basic principles of the latter are very similar to the former. Hence, in reverse docking, the ratio of the number of potential targets tested per individual test molecule is bigger than unity. In order to find new anti-cancer medicines, Zhang et al. [57] considered 902 distinct protein targets against 13 constituents of the herb *Brucea javanica*, thereby giving rise to 7119 possible constituent–target interactions. The targets were selected from known therapeutic targets of currently marketed commercial drugs. Since the screening of molecule–target interactions using previously reported experimental data and databases proved ineffectual, 52 of the 902 targets were selected for screening with the reverse docking against the 13 herb ingredients, involving a total of 492 target–ingredient interactions. Of the tested herb constituent–protein target interactions, 145 (covering 42 targets) of them had similar binding modes and comparable binding affinities to controls consisting of the current drug–target interaction. Zhang et al. [57] suggested that the so-called “promiscuity” of the herbal ingredients against multiple targets that they found in the docking study means that the herbal medicine is likely to be effective regardless of potential genetic variations between patients.

## 3. Combined Approaches Involving Molecular Docking

### 3.1. Static-Network-Based Approaches

**Figure 2:**
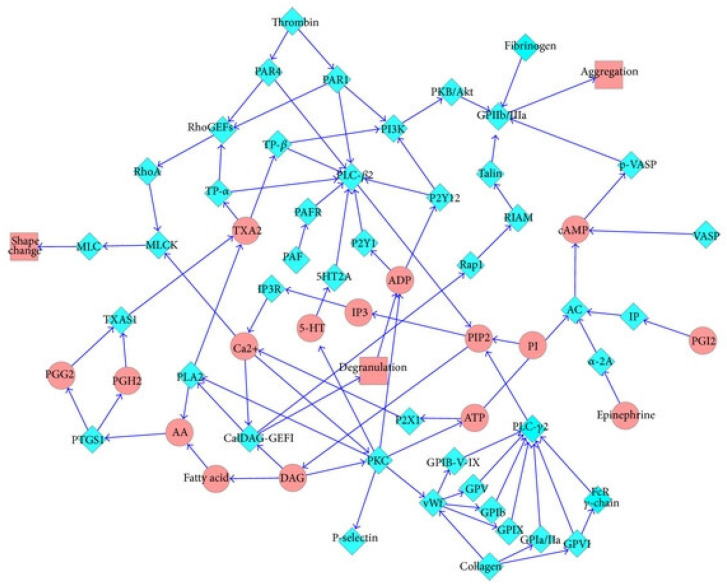
Pathway network of platelet aggregation. Blue diamonds and red ellipses represent proteins and small molecules, respectively. Reprinted with permission from Ref. [60]. Wiley, 2013.

Docking can be used as part of a network-based approach for an in silico prediction of the efficacy of compounds. This section will describe some examples. A network-based approach using docking was used to assess the efficacy of compounds for impacting the platelet aggregation pathways, as shown in Figure 2 [60]. Initially, a network was constructed from literature databases, where the enzymes important in the process of platelet aggregation were the nodes, and these included proteinase-activated receptor-1 (PAR1), PAR4, and Phospholipase-2 (PLA2). The connections between nodes (edges) were arrows, whose direction indicated downstream in the network, and each edge was given a weighting, initially a default value. Overall, the network consisted of 64 nodes and 91 edges (arrows). Nineteen of the enzymes in the network were chosen as targets for docking. Docking was performed on 413 compounds derived from Chinese herbs. Where a docking score was available for a particular target, it was used to determine the weight attached to all immediate connections arising from the corresponding node in the network if it exceeded the initial default value. The length of a path between nodes in the network was determined based on these weights, and the network efficiency was defined as the sum of the reciprocal lengths of the shortest path between each pair of nodes in the network. The network efficiency reflects the multi-target interaction of drugs. The impact of each compound on the network was assessed by the change in network efficiency using the docking scores for that compound to determine the edge weights. Overall, the effect of a compound on the network is considered more potent the more that the network efficiency decreases. The 40 compounds with the largest decreases in network efficiency were selected for experimental testing, and 19 of these were found to have antiplatelet aggregation activities, with the compounds silybin and papaverine found to be the most potent, and compared favorably with the then standard drug treatment for myocardial infarction, tirofiban. Further, the linear correlation coefficient between the decrease in network efficiency for a compound and the experimental results for blood anti-platelet aggregation activity was 0.67. However, if the impact on the network downstream of the target was included via the use of the network flux parameter, this correlation coefficient was improved to 0.73. However, the accuracy of the docking program, in determining compound affinity for a target, was found to affect the degree of correlation found. The importance of the network effect, where compounds bind to multiple inter-connected targets, was demonstrated by the fact that the correlation coefficients for single docking scores for test compounds and key protein targets versus experiment were lower than those for the network-based parameters.

Network-based approaches have also been coupled with docking simulations to elucidate the mechanism of the action of TCM formulations for type II diabetes (T2D) [56]. Consideration of the composition of the 11 herbs comprising the formulation for T2D was made using the Beilstein and Chinese Herbal Drug [61] databases, and 676 molecules were retrieved. Principal component analysis was used to show that these molecules were widely distributed in chemical space, and some were similar in structure to known drugs for T2D. These were then docked with 37 T2D-related proteins, such as the insulin receptor. Given that T2D is a complex disease involving many genes and gene products, the impact of targeting multiple proteins was assessed through network analysis. A drug–target (D–T) network was assembled where links were made between a given test molecule and a target protein if the docking score was in the top 3%. A drug–drug (D–D) network was assembled where links were made between test compounds if they shared one or more target proteins. In the D–T network, it was found that most molecules target a few proteins. The structure of the network was then assessed using several analysis methods. For example, the *k*-means method was used to show that the network had three major clusters and one small cluster. The smallest major cluster only consisted of the protein glucokinase and its drugs. However, a larger major cluster linked the two proteins glycogen synthase kinase-3 beta and protein kinase C, which are both important proteins in glycogen synthesis. A further larger cluster linked the glucagon-like peptide-1 receptor (GLP1R) and insulin degrading enzyme (IDE). This is probably because when GLP1R binds its agonist glucagon-like peptide-1, it increases insulin secretion, while IDE is a protease that cleaves insulin to maintain the homeostasis of insulin. Both the D–T and D–D networks were analyzed to determine the degree (number of interconnections) of each node corresponding to a test molecule. The nodes with the highest degree correspond to the most important molecules in the network that are also likely to have the greatest activity, and about 10–12 known active compounds were found to be amongst the 20 molecules with the highest degree.

The networks assembled through the analyses described above are often too complex for simple visual inspection to be useful, and so analysis algorithms are essential to extract the useful information contained therein.

### 3.2. Metabolic-Network-Based Approaches

Metabolic network models of biological systems consist of a set of ordinary differential equations that describe the enzymic catalysis in the network and the feedback inhibition or activation of the enzyme catalysts by their metabolites [16]. Metabolic networks are dynamic models that can simulate the perturbation of the network arising from the addition of exogenous compounds, such as from herbs. The feedback regulations and other pathways in the network mean that the effect of a particular molecule on the network, as a whole, may be very different to the effect of reaction of that molecule only at a single point in the network. A key issue with the use of metabolic networks is the ability to obtain values of the various kinetic parameters. The metabolic network model of a disease is that it represents a particular state of the network in which the production of disease-related molecules is abnormal [5]. The normal state is the state of the network desired after treatment. The aim of therapy is to shift the network back into the normal state. Algorithms, such as the Multi-Target Optimum Intervention (MTOI) method have been invented to identify the key set of several targets within a network and whether they each need inhibition or activation for a successful intervention. This identification is achieved through testing the impacts of various perturbations to the activities of potential targets suggested by a search algorithm, such as a generic algorithm, that ultimately aims to minimize the difference between the starting (disease) network state and the desired (normal) state [5]. The optimal solution can involve relatively mild impacts on individual enzyme activities made at multiple locations, leading to a greater overall effect on the whole network than a much larger single impact imposed at just one location. Side effects can be prevented through having multiple targets that can, between them, control the overall network balance [16].

The metabolic network approach has been used to assess the efficacy and mechanism of the action of herbal medicines for several diseases, namely, inflammation, HIV, and cancer [62,63,64]. Gu and Pei [65] have suggested a general workflow for testing herbal medicines using the computerized metabolic network method. First, the metabolic network is constructed using literature information and databases, such as the Kyoto Encyclopedia of Genes and Genomes (KEGG). This search is used to specify a group of ordinary differential equations (ODEs) that describe the network. It is then necessary to collect kinetic parameters for the ODEs describing the dynamics of the network. Where these are not directly obtainable from the literature, the set of ODEs can be used to predict the concentration curves of components in the network and these fitted to experimental data [5]. Docking simulations can also be used to quantify the interactions between compounds and proteins, using a predicted dissociation constant for each protein–ligand complex. Hence, the relevant protein structures must be found beforehand.

**Figure 3:**
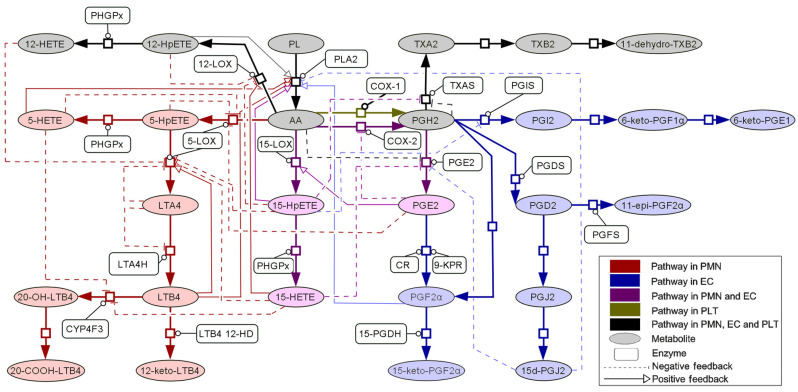
Metabolic network of AA in human PMNs, ECs, and PLTs. Reprinted with permission from Ref. [66]. American Chemical Society, 2015.

For example, inflammation processes are controlled by the arachidonic acid (AA) metabolic network (shown in Figure 3), and Lei and co-workers constructed a model of it consisting of a set of ODEs [16]. These equations simulate each time-dependent concentration of important enzymes [I], and molecules, in the network using a set of kinetic parameters collected from assays and computational prediction [9]. The original AA network model was that found in human polymorphonuclear leukocytes (PMNs), but this has been extended to AA metabolism in blood vessels as a whole, including three cell types, not just PNMs [66]. The models for the AA networks in the PMN, endothelial, and platelet cell types had 24, 29, and 11 ODEs, respectively, involving a total of 117 characteristic kinetic parameters [5]. The ODEs for the PMN represent 24 feedback loops, thus demonstrating the complexity of the network [67]. For example, the AA metabolic network consists of two main pathways with five key enzymes, namely, cyclooxygenase 1 and 2 (COX1/2), 5-lipooxygenase (5LOX), microsomal prostaglandin E synethase-1 (PGES), and leukotriene A4 hydrolase (LTA4H). Inflammatory syndromes can result from the overproduction of two metabolites, namely, prostaglandin E2 (PGE2) and leukotriene B4 (LTB4), within this network. For example, PGE2 is very associated with arthritis, while LTB4 is associated with coughs and asthma. Hence, the anti-inflammatory efficacy of a drug was judged by its ability to reduce the production of PGE2 and LTB4. Further, side effects from drugs for inflammatory syndromes are linked to the ratio of concentrations of prostacyclin (PGI2) and thromboxane A2 (TXA2), with the normal ratio being 0.68. If the ratio is too high, then the risk of gastrorrhagia is increased, as happens for aspirin (ratio ~5.2). If this ratio is too low, then cardiovascular risks are increased, as happens in the case of Vioxx (ratio ~0.28) [9]. The network model has been validated by comparing its predictions of the actions of a single COX-1 or 5-LOX inhibitor with observations [66].

The model can be used to simulate the action of inhibitors of different strengths, acting at different locations in the network. Simulations of the impact of single-target anti-inflammatory drugs (such as COX-1 inhibitors) have shown that these cannot stop the production of all inflammatory mediators [67]. However, intervention at both LT4H and COX can augment the 12/15-LOX and 15-LOX pathways, which produce endogenous anti-inflammatory agents [67]. Molecules that are more “promiscuous” and that target multiple locations in the network have a wider therapeutic window, even if they only have milder effects than molecules more specific for a single target, and thus the former are effective at lower plasma concentration [5]. Further, simulations with two inhibitors used in combination showed that the mixing ratio of the two makes big differences to the efficacy and safety of the mixture, and the relative inhibition constants of the two to each enzyme determines the overall therapeutic effect. In addition, a dual-functional single inhibitor molecule has been found to be more efficacious at a lower concentration than the combination of two separate, mono-functional inhibitors [5]. Hence, these findings suggested that the presence of more promiscuous compounds in herbal medicines would be more effective at lower doses. A single, multi-functional inhibitor also has a lower risk of drug–drug interactions that might cause side effects and will also be more robust against variations in plasma concentrations [7,66]. The presence of relatively promiscuous (and thus multi-functional) molecules in herbal medicines may also explain why they still work despite the range of concentrations of active ingredients that arise from harvesting at different times of year.

The AA metabolic network model was used to understand the efficacy and mechanism of action of anti-inflammatory TCM formulae [9]. It was assumed that the efficacy and side effects of a particular herb could be understood based upon its constituent molecules. Since the inhibition coefficients of most test molecules from TCM formulations for enzymes in the AA network are unknown, an all-to-all molecular-docking approach was used to obtain them. The overall workflow was as follows: Various TCM books were used to select 28 herbs that were recommended for use with inflammation-related syndromes, such as asthma and fever. The TCM database [68] was used to find out all known chemical compounds in these herbs. Then, steroid and glycoside compounds were removed from the list because, first, steroids are hormones that do not function in the AA network and thus may cause false positives, and, second, glycoside compounds are likely to be metabolized in the human body to remove glucose residues. This sifting left 237 remaining test molecules. Docking simulations of all the test molecules to the five key enzymes were used to obtain the “docking score” (Gibbs free energy) for each potential combination, and this was converted to the corresponding inhibition constant (*K_I_*). Then, the inhibitory effect of a given herb could be modelled as the sum of the effects of all its constituent test molecules using a variant of the Michaelis–Menten equation. However, it is difficult to know the likely plasma concentration that each molecule will achieve; so, it was assumed that each molecule would reach a value of 10 nM, which was set to be lower than expected for most drugs, and so a conservative estimate. The impact of plasma concentration was tested in a sensitivity study of this unknown parameter by randomly varying the plasma concentrations of the various components of a given herb mixture between values of 1 and 100 nM. It was found that this perturbation made little difference to the overall impact of the herb on the key pathways. This was interpreted to show how robust the final therapeutic effect of a herbal formulation is to variations in the mixture composition or the concentration of active ingredients in herbs due to temporal variation in harvesting, etc. As mentioned above, the AA network consists of the PGE2 and LTB4-producing pathways. An individual herb was ranked according to its ability (assessed by multiplying [I]/*K_I_* values in the same pathway) to eliminate PGE2 or LTB4. Via this assessment, the (mixture of compounds corresponding to the) herb *Glycyrrhiza uralensis* was found to have the best inhibition of both PGE2 and LTB4, which is consistent with its traditional reputation as applicable to many inflammatory syndromes [9]. However, most of the herbs tested preferentially reduced LTB4 production, rather than PGE2 production. Meng et al. [66] suggested that may be because most of the herbs had been most often traditionally selected to treat asthma or coughs. In general, it was found that different test compounds in the same herb or herb formulation tended to have different targets, with the possibility of covering almost the whole AA network to achieve a superior therapeutic effect. Further, some combinations of herbs also had a synergistic effect. For example, the combination of *Forsythia suspensa* and *Scutellaria baicalensis* had a total inhibition of PGE2 (of 27%), which was higher than the sum of their individual numbers (20%). In addition, the same overall therapeutic effect (inhibition level) could be obtained with lower plasma concentrations of test compounds when these were in combinations corresponding to multiple herb formulations compared to just individual herbs. This may suggest how formulations of several herbs can lead to lower side effects than for single herb medicines because lower doses of the former are needed.

It is suggested [65] that the lack of topological information can lead to the failure of metabolic networks and that, in such circumstances, Boolean network modelling may be an alternative. Wang et al. [69] suggested that Boolean networks might be used when the large wealth of quantitative kinetic data needed for metabolic network modeling is not available by experiment and/or docking. In the absence of quantitative kinetic data, a Boolean model can still model some dynamic aspects of biological systems, such as state transitions. A Boolean network consists of a set of nodes whose state is binary and is determined by other nodes in the network. Hence, such a network model lies between the static and continuous dynamic (metabolic) in complexity. The Boolean network might be amenable when the activity level of a biological entity varies more in a stepwise function of concentration. Since Boolean models do not explicitly incorporate the potentially wide-ranging individual kinetics of separate entities, the resultant dynamics can be highly sensitive to the more abstract updating scheme used in Boolean network operation. For a system where a suitable updating scheme is not feasible, a metabolic network model is, thus, required. In addition, a compromise model consisting of a combination of Boolean elements with differential equations is possible for some systems and requires fewer kinetic parameters than the full metabolic model [69]. However, Boolean models may be less applicable to modeling the impacts of herbal medicines because their effects are often continuous, partial, or middling rather than more discrete step changes associated with bridging defined thresholds, and the influences of herbal medicines can arise over many quite different time scales, such as short-term effects contrasting with those building with long-term treatments.

### 3.3. Combination of Molecular Docking with Common Pharmacophore Matching

**Figure 4:**
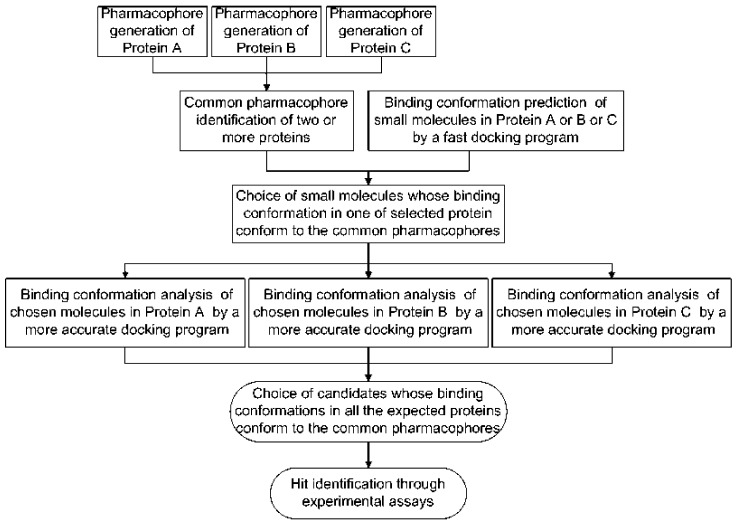
Strategy of multi-target inhibitor discovery. Reprinted with permission from Ref. [70]. ACS, 2008.

The development of software for automating methods of the construction of 3D pharmacophores has enabled a general approach that can be used for screening for multi-target inhibitors, both from synthetic sources and herbal medicines, involving combining molecular docking with common pharmacophore matching, as shown in Figure 4 [70]. The combination of pharmacophore methods allows the direction of the docking simulations with pharmacophore templates and can thus speed up the overall screening of a set of compounds via docking [71]. Further, while pharmacophore screening alone can end up with a mixture of both weak and strong ligands, the combination with docking enables the strong ligands to be selected out. In particular, the combined approach can make screening many test molecules against multiple targets a feasible goal. While the combined method speeds up screening, it can potentially unduly limit the number of molecules identified since the more complex the set of pharmacophores, the more restricted will be the set of compounds with the required features.

One version [70] of the combined method involves, first, finding the sets of molecular structural features that are recognized at each binding site responsible for the biological activity of the relevant target protein(s) to develop pharmacophore models for each target. Second, the approach then identifies common pharmacophores by comparing the individual models for each site if these exist. Third, a rapid docking algorithm is used to predict the binding confirmation of all test molecules in one of the target proteins, and then those molecules whose binding configuration can accommodate the common pharmacophore identified in the second step are selected out. Fourth, the binding configurations of these initially selected-out compounds in the other target proteins are found with a more rigorous docking simulation, and the set of selected compounds is further refined to molecules where their binding configurations in all target proteins can accommodate the common pharmacophore model. The resultant, further refined set of molecules may each have relatively low affinities across all of the targets, but, as already mentioned above, the combined therapeutic effect of a given molecule at several locations across biological networks may be cumulatively larger than a single molecule with a greater affinity at just one point in the network. The combined docking and pharmacophore approach has been validated on synthetic drugs [70] but might also be applied to compounds occurring in herbal medicines.

Ehrman et al. [71] conducted a combined pharmacophore and docking screening for multi-target anti-inflammatories in Chinese herbs and their combined formulations. The multiple protein targets were cyclo-oxygenases 1 and 2 (COX1/2), p38 MAP kinase (p38), c-Jun terminal-NH_2_ kinase (JNK), and type 4 cAMP-specific phosphodiesterase (PDE4). These proteins had been previously (in the literature) found to play roles in a variety of inflammatory syndromes [71]. Further, previously, it had been found that the PDE4 inhibitor roflumilast also prevents the phosphorylation of both p38 and JNK, thus blocking the production of inflammatory mediators such as TNF-α and interleukin (IL)-1β. Ehrman et al. [71] proposed that this finding suggests that molecules with the ability to inhibit more than one of these targets have greater potential for treating complex inflammatory syndromes. These workers also suggested that multi-target therapy is easier to achieve with a mixture of molecules, rather than using a single scaffold, since greater chemical diversity is possible with the former. Ehrman et al. [71] used multiple pharmacophore models of the four protein targets to screen 5978 compounds from their database of constituents of Chinese herbs that had passed initial screening for drug-like properties via the Lipinski “rule-of-five”. The resulting suggested hits were then submitted for screening with docking software. The types of phytochemical classes that were found to be most involved in inhibiting inflammatory targets were phenolics, including lignans and flavonoids, and smaller terpenoids, such as monoterpenes, iridoids, and sesquiterpenes. Overall, it was found that 48% of 100 herbs tested are likely to have inhibitors for two or more targets, and 14% of herbs had more than one inhibitor for a single target that also came from different types of the aforementioned phytochemical classes.

The reverse-docking study of Zhang et al. [57] used a parallel pharmacophore approach to independently validate the findings obtained from docking. It was found that of the 52 herb constituent–protein target pairs highlighted by the docking study, all contained at least one common pharmacophore feature, and 37 of the target proteins shared at least three common pharmacophores.

## 4. Conclusions

The underlying philosophy of herbal medicines, such that they contain multiple active components that often may have only middling affinity but for multiple targets relevant to a given disease state, has been seen to be consistent with the recent multiple-target strategy for developing new effective drug treatments for complex diseases. Hence, given the realization of the need to target multiple sites in biological networks to perturb a disease state back into the healthy one, this has spawned many studies into SAM effects due to herbal medicines, including both single herbs and multi-herb formulae. Molecular docking enables some understanding of the underlying mechanisms of SAM effects to be discerned, as multiple molecules from a given herbal mixture can be docked with various potential targets to determine affinities. However, molecular docking has also been shown to be a critical component in several of the new systems biology approaches. Molecular docking can provide quantitative weightings for the connections within static (molecule-target) networks, or supply estimates of kinetic parameters for use in dynamic metabolic networks. Molecular docking can also be combined with pharmacophore modeling to provide a hybrid method that greatly improves the efficiency of screening. Overall, molecular docking has been shown to be a highly useful tool to aid in the provision of evidence for the efficacy of herbal medicines, previously only supported by traditional usage.

## Figures and Tables

**Table 1:** Molecular-docking studies of synergistic, additive, or multi-target (SAM) effects in herbal medicines. (ns = not specified; Y = yes; N = no).

Disease Target	No. of Molecular Targets	No. of Herbs	Total no. of Test Compounds	Docking Software/ Scoring Type	Outcome of Docking	Use of Systems Biology Tools?	SAM Effect?	Reference
Alzheimer’s disease	1	1	3	GOLD/empirical	1′,2′-dihydroxy-3′-pentadec-8-enylbenzene and 1′,2′-dihydroxy-3′-pentadeca-8,11-dienylbenzene from *Semecarpus anacardium* inhibited acetylcholinesterase (AChE)	N	Y	[33]
Alzheimer’s disease	1	5	2	AutoDock Vina-1.1.2/knowledge-based and empirical	Palmatine and berberine, components of a methanolic extract of *Tinospora cordifolia*, both showed inhibitory effects on AChE	N	Y	[34]
Aspergillosis	3	1	1	patchDock/geometric fit and atomic desolvation energy	“Strong” binding of eucalyptol to all fungal cell receptors	N	Y	[35]
Cancer	4	1	2	AutoDock/knowledge-based and empirical	Withanone (Wi-N) and Withaferin A (Wi-A), in Ashwagandha, both bind strongly to mortalin and p53, while Wi-A also bound to 2 other anti-cancer targets	N	Y	[36]
Cancer	1	1	7	AutoDock/knowledge-based and empirical	Trichloromethyl 9-anthracenecarbodithioate and 4H-1-benzopyran-2-carboxylic acid, 5-amino-6-hydroxy-4-oxo-ethyl ester from *Potentilla nepalensis* both showed similar binding affinity with glycogen synthase kinase 3β as the standard synthetic drug	N	Y	[37]
Cancer	2	1	2	AutoDock Vina-1.2.3/knowledge-based and empirical	Rosmarinic acid and cholorogenic acid from *marrubium lutescens* extract show prominent inhibition of MARK4 and TYRP1 targets	N	Y	[38]
Cardiovascular disease	8	11	501	LigandFit (in Cerius2)/force field	100 compounds had interactions with more than one target, and manninotriose from *Rehmannia glutinosa* had interactions with 3 different targets	N	Y	[39]
COVID-19	1	10	25	AutoDock Vina/knowledge-based and empirical	Binding affinities of all compounds all very similar (~5 kcal/mol) and relatively weak	N	Y	[40]
COVID-19	1	1	56	AutoDock Vina, GOLD, and MOE/knowledge-based and empirical	5 compounds from essential oil of *Thymus schimperi* were top hits; 2 with good ADMET profiles	N	N	[41]
COVID-19	2	1	18	MOE 2015.10/force field	Synergistic interactions of 17 out of 18 compounds that constitute 99.4% of garlic essential oil	N	Y	[42]
COVID-19	6	84	171	Molegro Virtual Docker v.6.01/force field	Individual molecular affinities for all targets were all weak and thus unlikely to interact with viral targets	N	N	[43]
Drug-adverse interactions	5	1	12	Accelrys Discovery Studio 3.1/ns	All 12 compounds from Ginger tested interacted with human CYPs, but CYP2D6 had largest interactions	N	Y	[44]
Endocrine disruption	2	0	82	AutoDock Vina/knowledge-based and empirical	Eucalyptol, Dihydro-β-Ionone, and (-)-α-pinene showed 20–40% inhibition of dehydroepiandrosterone production	N	Y	[45]
Gastro-intestinal and inflammatory syndromes	1	1	7	AutoDock/knowledge-based and empirical	Isovitexin from *Gentiana lutea* root extract binds strongly to MEK1 protein	N	N	[46]
Hyperlipidemia	6	3	21	Vina and DOCK6/Consensus	β-sitisterol, bis-desmethoxycurcumin, cucurbitacin D and E, myricetin, phloretin, quercetin, and rutin from *Curcuma xanthorrhiza, Sedium edule, Syzigium polyanthum*, multi-target HMGCR, PPARA, AKTI, EGFR, MMP9, and TNF but with only HMGCR targeted by all 8 compounds.	Y	Y	[31]
Inflammatory syndromes	1	1	2	LigandFit/force field	2 saponins from *Parthenium hysterophorus* found to be potent inhibitors of TNF-α	N	Y	[47]
Inflammatory syndromes	1	1	65	MOE 2016.08 and AutoDock/force field and knowledge-based and empirical	4-((1E)-3-hydroxy-1-propenyl)-2-methoxyphenol, 2-flurobenzoic acid 1-adamantylmethyl ester, 3-(3′,5′-dimethoxy-4′-hydroxyphenyl)-2-propen-1-ol, and (E)-3,3′-dimethoxy-4,4′-dihydroxystilbene from *Habenaria didtata* showed high binding affinities and synergistic effect	N	Y	[48]
Inflammatory syndromes	2	1	20	PyRx-AutoDock/knowledge-based and empirical	Stigmastan-3,5-diene, phthalic acid, and 3-alpha-hydroxy-5,16-androstenol from *Portulacaria afra* had high binding affinities for COX-1/2	N	Y	[49]
Influenza	1	ns	20,000	LigandFit/force field	Rosmaricine_5 and rosmaricine_16 from *Rosmarinus officinales* have highest binding energy with homology model of H1N1 hemaglutinin	N	Y	[50]
Non-alcoholic fatty liver	10	1	19	GLIDE/empirical	Most saponins from black form of *Panax ginseng Meyer* had good binding affinity with AKT1	Y	Y	[51]
Obesity	1	1	5	AutoDock Vina-1.5.6/knowledge-based and empirical	Ginkgetin, sciadopitysin, bilobetin, isoginkgetin, and quercetin from *Ginkgo biloba* all bound tightly to human pancreatic lipase	N	Y	[52]
Osteoarthritis	13	2	227	LigandFit (in Cerius2)/force field	From *Chuanxiong Rhizome-Paeonia abifora Pall* herbal combination, 39 compounds interacted with more than one target, and some, such as eugeniin, interacted with 3 targets	Y	Y	[53]
Pneumonia	7	2	7	AutoDock Vina/knowledge-based and empirical	7 compounds from *Astrgali radix* and *Atractylodis macrocephalae* rhizomes active as comparable synthetic drug standards on targets	Y	Y	[54]
Rheumatoid arthritis (RA)	36	1	30	GLIDE/empirical	4 flavonoids from *Alpina calcarata* have higher binding affinity for RA targets than other constituents	Y	Y	[26]
Tuberculosis	1	1	40	GLIDE/empirical	Withanolide E, F, and D and Withaferin-diacetate-2-phenoxy-ethy-carbonate identified as potential inhibitors of PknG	N	Y	[55]
Type II diabetes (T2D)	37	11	676	LigandFit/force field	Overall hit rate of 35%	Y	Y	[56]

## Data Availability

Not applicable.

## Appendix A. Search Methodology

Sources were obtained from keyword search (herb* AND synerg* AND docking; herbal medicine AND side effects; docking AND synerg*) in the Web of Science database. Further sources found in the initial sources were then followed up by cited reference search.
